# Cancer statistics, 2026: Charting a course for a national cancer research agenda

**DOI:** 10.3322/caac.70061

**Published:** 2026-01-13

**Authors:** Primo N. Lara, Dawn L. Hershman

**Affiliations:** ^1^ University of California Davis Comprehensive Cancer Center Sacramento California USA; ^2^ Herbert Irving Comprehensive Cancer Center, Columbia University Medical Center New York New York USA

In this issue of *CA: A Cancer Journal for Clinicians*, the American Cancer Society (ACS) delivers its highly anticipated annual portrait of the nation's cancer burden.^1^ As in previous years, this authoritative and widely cited report draws on data from every state and the District of Columbia, aggregating data from cancer registries and the National Center for Health Statistics to provide the most comprehensive and detailed view of cancer incidence, mortality, demographics, and disparities.

In 2026, the ACS estimates that there will be more than 2.1 million new cancer diagnoses (or about 5800 cases daily) in the United States. In men, the most common malignancies continue to be prostate, lung, and colorectal cancers; in women, breast, lung, and colorectal cancers are most common. Yet this year's projections also bring some troubling trends: more men with prostate cancer will be diagnosed at advanced stages, in which a cure is less attainable; breast cancer incidence continues to rise, especially among women younger than 50 years; and colorectal cancer is also increasing in adults younger than 50 years.

Counterbalancing these concerns is a profoundly encouraging trend: cancer mortality in the United States continues its long, steady decline. Since 1991, cancer death rates have fallen by 34%, translating to 4.8 million lives saved. This extraordinary progress reflects decades of iterative and transformative advances in cancer research and the implementation of those findings into prevention, screening, and clinical care. In short, research saves lives!

In the case of publicly funded clinical trials conducted through the National Cancer Institute's (NCI's) National Clinical Trials Network (NCTN), this progress has also been remarkably cost effective. In a study of NCTN trials since 1980, approximately 14.2 million additional life‐years were gained by patients with cancer, with projected gains of 24.1 million life‐years by 2030. Incredibly, the federal investment cost per life‐year gained was estimated to be just $326.^2^

These ACS projections have provided a rigorous, empirical foundation for shaping, prioritizing, and implementing research strategies across both laboratory and clinical settings. They also clarify where our knowledge falls short and expose where cancer health disparities persist. In spotlighting malignancies with rising incidence, stubbornly high mortality, or persistent inequities, these data direct researchers to focus efforts on the most urgent unmet needs in cancer research, ensuring that progress will have a profound impact on patients and communities.

Within the United States research ecosystem of federally funded clinical trials, the NCI's NCTN, Experimental Therapeutics Clinical Trials Network (ETCTN), and Cancer Centers Program all play pivotal roles in translating the ACS's cancer statistics into actionable research. Within the NCTN cooperative groups, the research bases (SWOG Cancer Research Network, the Eastern Cooperative Oncology Group‐American College of Radiology Imaging Network Cancer Research Group, the Alliance for Clinical Trials in Oncology, the NRG Oncology Foundation, and the Children's Oncology Group) provide a platform for conducting practice‐changing, investigator‐initiated phase 2 or 3 clinical trials, aligned with emerging scientific research priorities.^3^ Through the NCI's National Community Oncology Research Program, trials extend into the community setting, enabling the enrollment of participants who reflect the evolving demographics of the US cancer population.^4^

Within the NCTN, there is a commitment to pursuing a balanced portfolio of clinical trials that highlight the strength of federally funded research and a focus on areas that are not prioritized by industry‐sponsored research. These include pragmatic trials with simplified designs, liberal eligibility criteria, and enhancements that reduce the burden of data collection; comparative effectiveness trials that randomize individuals to competing standards of care; multimodality studies that combine systemic therapies with surgical and/or radiation approaches; trials in rare tumor subsets; and treatment de‐escalation strategies, among others.^4^, ^5^, ^6^ In a recent analysis comparing federally and industry sponsored cancer clinical trials over the past 2 decades, federally sponsored trials were more likely to investigate nontreatment interventions; to examine complex, multiagent and multimodality treatment regimens; to evaluate treatments in rare cancers and pediatric populations; and to incorporate de‐escalation designs. These findings underscore the critical role of federal sponsorship in the conduct of clinical cancer research.^7^

Similarly, the ETCTN uses ACS cancer statistics in the context of molecular phenotypic data to identify which molecular targets or uncommon malignancies merit investment in first‐in‐man and signal‐finding studies, ensuring that early phase development is aligned with population‐level priorities as well as scientific innovation.

The NCI's Cancer Centers Program provides the intellectual and translational infrastructure that ties these networks together. With mandates in high‐impact basic, clinical, and population science research, community outreach and engagement, and education/training initiatives, NCI‐designated centers incorporate national cancer statistics and catchment area data to define regional research priorities and clinical care delivery strategies. These insights guide overall center strategy, pilot funding for research/recruitment investments and multidisciplinary research programs, and they stimulate leadership in NCTN/ETCTN trial development. Because many Cancer Centers serve as hubs for cooperative group leadership, their insights directly shape national trial portfolios.

Clinical trialists have long depended on the comprehensive information and trends within the annual ACS report to assess trial feasibility, anticipate patient selection, and define appropriate trial end points. For instance, incidence data for common cancers, such as prostate, breast, lung, and colorectal malignancies, may prompt the development of prevention, screening, or interception studies, whereas high‐mortality cancers, such as pancreatic, liver, and certain lung cancers, often motivate innovative early phase trials exploring therapies aimed at aggressive tumor biology. In addition, insights into health disparities across socioeconomic, geographic (e.g., rural vs. urban), and demographic factors (e.g., age, sex, race) help inform trial designs that prioritize populations requiring targeted recruitment strategies and strengthened community engagement. Moving forward, increased national attention should focus on the reasons behind the rise of early onset malignancies, the differences in tumor biology and treatment effects, and the long‐term consequences of the growing number of young survivors of cancer.

In summary, the ACS's annual cancer statistics report serves as one of the cornerstones of the nation's federally funded research enterprise. It provides a robust pathway that bridges the population‐level cancer burden and the full continuum of scientific discovery that has contributed to the progress and impact of reduced lives lost to cancer. By guiding critically important federal investments, drug/biomarker development, and clinical trial priorities to the areas of greatest need, this report ensures that resources are directed where they can deliver the greatest improvement in patient outcomes and public health.

## CONFLICT OF INTEREST STATEMENT

Primo N. Lara Jr and Dawn L. Hershman disclosed no conflicts of interest.
